# Behavioural responses to human‐induced change: Why fishing should not be ignored

**DOI:** 10.1111/eva.12456

**Published:** 2017-02-07

**Authors:** Beatriz Diaz Pauli, Andrew Sih

**Affiliations:** ^1^ Department of BiologyUniversity of BergenBergenNorway; ^2^ Department of BiosciencesCentre for Ecological and Evolutionary Syntheses (CEES)University of OsloOsloNorway; ^3^ Inst. d'Ecologie et des Sciences de l'Environnement – Paris (iEES‐Paris)Sorbonne Universités/UPMC Univ Paris 06/CNRS/INRA/IRD/Paris Diderot Univ Paris 07/UPEC/ParisFrance; ^4^ Department of Environmental Science and PolicyUniversity of CaliforniaDavisCAUSA

**Keywords:** animal personalities, behaviour, correlated traits, evolution, fisheries management, fishing selection, predation

## Abstract

Change in behaviour is usually the first response to human‐induced environmental change and key for determining whether a species adapts to environmental change or becomes maladapted. Thus, understanding the behavioural response to human‐induced changes is crucial in the interplay between ecology, evolution, conservation and management. Yet the behavioural response to fishing activities has been largely ignored. We review studies contrasting how fish behaviour affects catch by passive (e.g., long lines, angling) versus active gears (e.g., trawls, seines). We show that fishing not only targets certain behaviours, but it leads to a multitrait response including behavioural, physiological and life‐history traits with population, community and ecosystem consequences. Fisheries‐driven change (plastic or evolutionary) of fish behaviour and its correlated traits could impact fish populations well beyond their survival per se*,* affecting predation risk, foraging behaviour, dispersal, parental care, etc., and hence numerous ecological issues including population dynamics and trophic cascades**.** In particular, we discuss implications of behavioural responses to fishing for fisheries management and population resilience. More research on these topics, however, is needed to draw general conclusions, and we suggest fruitful directions for future studies.

## 1 Introduction

Aquatic ecosystems have always experienced environmental change, but human activities have greatly accelerated such change (Halpern et al., 2008). Human activities have led to decline and even extinction of many populations. Increasing evidence shows that populations are responding to the novel human‐induced selection by modifying ecologically relevant traits (Hendry, Farrugia, & Kinnison, 2008). In recent years, it has become evident that variation in behavioural responses is a key for explaining whether species adjust to the environmental change, thrive or succumb (Sih, Ferrari, & Harris, 2011). Behavioural responses to human disturbance range from initial plastic responses to evolutionary ones and have been reviewed elsewhere (Candolin & Wong, 2012; Sih et al., 2011; Smith & Blumstein, 2013; Tuomainen & Candolin, 2011; Wong & Candolin, 2015).

Notably, however, behavioural effects of harvesting, particularly fishing, have been largely ignored (but see Miller, 1957; Heino & Godø, 2002; Uusi‐Heikkilä, Wolter, Klefoth, & Arlinghaus, 2008; Smith & Blumstein, 2013 for brief discussions) and are only recently getting more attention (Arlinghaus et al., 2016). This is unfortunate, because fishing is a critically important source of mortality in most fish stocks. Life‐history traits are believed to be the main target of fishing selection (reviewed by Heino, Diaz Pauli, & Dieckmann, 2015), but harvesting is likely also driving the evolution of fish behaviour (Arlinghaus et al., 2016; Uusi‐Heikkilä et al., 2008). Studying the behavioural response to fishing and its correlated physiological and life‐history traits allows us to better understand the implications that fishing‐induced changes have for fish populations and management.

Fishing‐induced selection affects any trait that regulates an individual's vulnerability to fishing (i.e., survival). Fishing could lead to plastic changes in behaviour through developmental plasticity and learning, or evolutionary changes if the individual differences in behaviour linked to vulnerability are heritable. Fishing can also alter behaviour through effects of fishing‐induced changes in life history and correlated behaviours. Fishing‐induced change can concur, affect or counteract changes due to natural selection and other selective forces, and their interplay ultimately determines the direction and intensity of the evolutionary change (e.g., Edeline et al., 2007). The resulting phenotype change (plastic or evolutionary) may impact populations, communities and ecosystem (Arlinghaus et al., 2016; Palkovacs, Kinnison, Correa, Dalton, & Hendry, 2012). Despite the awareness decades ago that fishing could select for certain behaviours (Miller, 1957), formally studying behavioural selectivity of fishing and its ecological and evolutionary consequences has been, until recently**,** scarce.

Here, we (i) compile studies to present how different fishing methods (active and passive gears) are selective towards behavioural traits and (ii) discuss the population, community and ecosystem level consequences of fishing‐induced changes in behaviour. We complement Arlinghaus et al.'s (2016) review on effects of passive fishing gear on behaviour by considering the effect of active gears such as trawls and the indirect effects of fishing on behaviour when it is not the target trait. Moreover, we compare the ecological consequences of a multitrait (behavioural, physiological and life history) response to fishing versus the consequences expected when only life‐history traits are taken into account. Future experiments are encouraged to study the behavioural and multitrait response to active gears to obtain a more complete view of the effect of fishing on the exploited populations.

## 2 Fishing‐induced selection on behaviour

Passive gears rely on fish diel and seasonal movements, and feeding behaviours (when bait is present) during the capture process. Passive fishing gears involve the capture of fishes by entanglement, entrapment or angling devices and hence rely on the target species to move towards the gear, while active fishing gears are moved by humans or machines in pursuit of the target (Gabriel, Lange, Dahm, & Wendt, 2005). Active gears have been thought to catch all individuals present in front of the trawl or seine mouth (Walsh, 1992). However, both trawls and seines allow individuals to escape the gear, either avoiding the gear, finding escape routes or during slipping of the seine (i.e., release of part of the catch over the headline right before the fish is drawn aboard; Engås & Godø, 1989; Misund, 1990; Kelleher, 2005; Heino et al., 2011). Fishing selection is well known to have direct and indirect effects on life‐history traits (Heino et al., 2015). For example, size‐selective fishing can directly favour slow growth (e.g., Conover & Munch, 2002) or early maturation resulting in indirect lower investment in growth during adulthood (Heino et al., 2015). Similarly, we expect fishing to be both directly and indirectly selective towards behavioural traits resulting in a multitrait response and that passive and active gears would affect behaviour differently. Thus, we review these separately in the following sections.

## 3 Fishing direct selection on behaviour

### 3.1 Passive gear

Early studies showed population and individual differences in angling vulnerability of several species (see Miller, 1957 for an early review). Arlinghaus et al.'s (2016) recent review concluded that boldness seems to be the behavioural trait correlated to angling vulnerability due to both selection and plasticity; for instance, bold individuals are angled more often in carp (*Cyprinus carpio*; Klefoth, Skov, Krause, & Arlinghaus, 2012), largemouth bass, smallmouth bass (Suski & Philipp, 2004) and brown trout (Härkönen, Hyvärinen, Paappanen, Vainikka, & Tierney, 2014). Male largemouth (*Micropterus salmoides*) and smallmouth bass (*M. dolomieu*) were more vulnerable to angling while guarding their nests (Suski & Philipp, 2004), and although boldness was not directly tested for vulnerability, guarding males were more aggressive, which is commonly associated with boldness (Sih, Bell, & Johnson, 2004). Vulnerability to angling in brown trout (*Salmo trutta*) was associated with exploration, which is also related to boldness (Härkönen et al., 2014). Along these lines, numerous studies have associated plastic changes due to learning or reduced willingness to forage with a decrease in vulnerability after being hooked by angling (reviewed in Miller, 1957 and Arlinghaus et al., 2016). Angling's higher selectivity towards bold individuals results in a skewed distribution towards shy individuals in populations exposed to intense fishing pressure (a timidity syndrome; Arlinghaus et al., 2016), as seen for instance in the wild for painted comber (*Serranus scriba*), amago salmon (*Oncorhynchus masou ishikawae*) and some coral reef fishes (Alós, Palmer, Trías, Díaz‐Gil, & Arlinghaus, 2015; Bergseth, Williamson, Frisch, & Russ, 2016; Januchowski‐Hartley, Graham, Cinner, & Russ, 2015; Tsuboi, Morita, Klefoth, Endou, & Arlinghaus, 2016).

**Figure 1:**
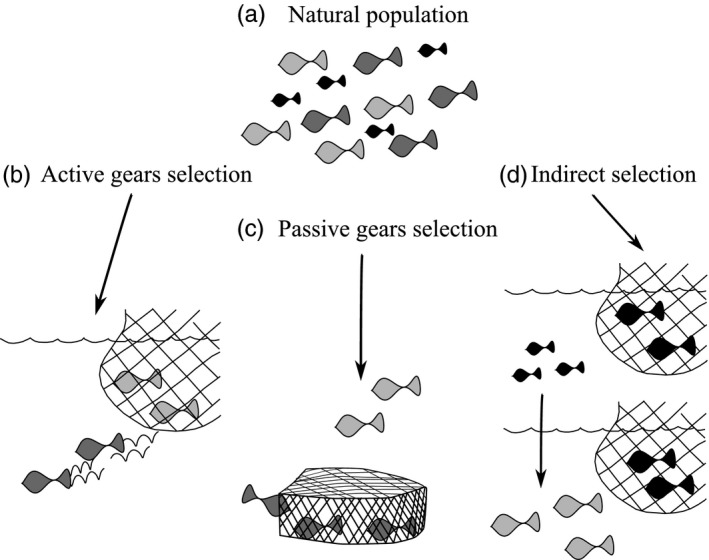
Conceptual diagram showing how fishing can affect the natural distribution of behavioural types in the population (a). Passive (b) and active (c) gears directly target different behavioural types, for example bold individuals represented as dark grey and shy individuals light grey. Behavioural composition also can be altered indirectly (d) by size‐selective harvesting if physiological, behavioural and life‐history traits are correlated

Although the timidity syndrome seems intuitive, angled bluegill sunfish (*Lepomis macrochirus*) were shy and not bold; this discrepancy could be explained because angling took place close to refuge areas where shy individuals are more common (Wilson et al., 2011). Also, angling vulnerability in perch (*Perca fluviatilis*) was not related to boldness (Kekäläinen, Podgorniak, Puolakka, Hyvärinen, & Vainikka, 2014; Vainikka, Tammela, & Hyvärinen, 2016), but rather associated with exploration (Härkönen, Hyvärinen, Niemelä, & Vainikka, 2015). These differences in results show that the link between behaviour and vulnerability to angling is related to the ecology of each species, and also to differences in experimental set‐ups used. Angling might result in an increase in boldness in the population when vulnerability to passive gears is independent of size and other traits (Jørgensen & Holt, 2013), but this is not common, and hence, the general pattern might be an increase in timidity in the population which could be due to either a plastic or evolutionary change (Arlinghaus et al., 2016; Table 1, Figure 1c).

**Table 1:** Expected behavioural types under selection for each fishing method

Fishing method	Fishing type	Selected behavioural type	References
Angling	Passive	Bold	Alós, Palmer, et al. (2015), Klefoth et al. (2012), Suski and Philipp (2004) and Tsuboi et al. (2016)
Pots and traps	Passive	Bold*; Exploration and willingness forage*	Diaz Pauli et al. (2015), Ovegård et al. (2012) and Wilson et al. (1993)
Gill nets	Passive	Bold, Active*; Willingness forage*	Biro and Post (2008), Olsen et al. (2012) and Ovegård et al. (2012)
Trawl	Active	Swim upwards^a^, constant swimming and shyness, shallow‐water habitat preference	Alós et al. (2014), Jakobsdóttir et al. (2011), Killen et al. (2015), Kim and Wardle (2003), Opdal and Jørgensen (2015) and Underwood et al. (2015)
Seine	Active	Probably shy, but not conclusive	Moav and Wohlfarth (1970) and Wilson et al. (1993, 2011)

Italics refer to behaviours affecting encounter rate, not catchability.

a Probably only applicable for species with tendency to escape downwards; e.g., for cod, yellow flounder.

Vulnerability to gill nets and pots is associated with high activity, boldness or short habituation times in rainbow trout (*Oncorhynchus mykiss*; Biro & Post, 2008) and pumpkinseed sunfish (*Lepomis gibbosus*; Wilson, Coleman, Clark, & Biederman, 1993), in experimental ponds. In guppies (*Poecilia reticulata*), vulnerability to being caught by an unbaited trap depended on their exploratory behaviour in laboratory conditions (Diaz Pauli, Wiech, Heino, & Utne‐Palm, 2015). Active individuals encounter the trap more often, but it was exploratory behaviour that was associated with the trapping event; more exploratory individuals were trapped more often relative to nonexploratory fish.

The association of behavioural types with capture by passive gear can come via various mechanisms in the wild. Olsen, Heupel, Simpfendorfer, and Moland (2012) showed that trapping, angling and gillnetting selectively removed Atlantic cod (*Gadus morhua*) that occupied shallow waters and displayed extensive diel vertical migration and consistent horizontal movements; these behaviours might be associated with being bold. Quinn, Hodgson, Flynn, Hilborn, and Rogers (2007) showed that angling and gillnetting are selective towards early migration in salmon (*Oncorhynchus nerka*). Boldness is often linked to dispersal and migration in fish, as bold individuals move longer distances (Fraser, Gilliam, Daley, Le, & Skalski, 2001) and are more prone to migrate (Chapman et al., 2011). Ovegård, Berndt, and Lunneryd (2012) found that baited gears (traps and hooks) captured lower condition cod, relative to gill nets. Although they did not directly assess fishing selectivity towards behaviour, they concluded that pots and hooks might selectively capture more bold individuals, which actively make the choice of entering traps; while gill net capture depends on higher activity in individuals in better condition, it is independent of the active choice of bold individuals to approach the gear (Ovegård et al., 2012). Overall, Ovegård et al. (2012) and Diaz Pauli et al. (2015) suggest that vulnerability towards a pot or trap requires boldness and willingness to explore and enter the gear rather than just high activity, which is related to gear encounter (Table 1, Figure 1c). Whether exploration measured in laboratory conditions correlates with active decisions to enter a pot in natural conditions remains to be tested.

### 3.2 Active gears

The first response towards a trawl is diving as seen in cod from video recordings and tracking devices in natural conditions (Handegard & Tjøstheim, 2005; Rosen, Engås, Fernö, & Jørgensen, 2012), and after experiencing the trawl once, vulnerability to trawling is reduced by leaving the area and avoiding the vessel (Pyanov, 1993). More details of fish behaviour are provided by small‐scale experiments. Fish initially avoid penetrating meshes, but the probability of passing through a mesh increased with prior experience (Brown & Warburton, 1999a; Özbilgin & Glass, 2004). However, escape latency substantially differs among species and depends on the location of the escape route (Hunter & Wisby, 1964). The species‐specific escape route is also evident in natural conditions; haddock (*Melanogrammus aeglefinus*) tend to escape a trawl over the headline, while cod seem to actively seek openings at the bottom (Engås & Godø, 1989; Walsh, 1992). Little is known, however, about behavioural differences between escapees and captured individuals. Sociability seems to play a role, as groups of fish were better at escaping than pairs or singletons (Brown & Warburton, 1999b; Hunter & Wisby, 1964) and explorer/bold individuals were better at escaping than nonexplorers/shy ones (Brown & Warburton, 1999a; Diaz Pauli et al., 2015). Swimming and metabolic performance might also be associated with vulnerability to trawl. In minnows (*Phoxinus phoxinus*), individuals with higher anaerobic capacity and burst swimming performance were less vulnerable to a trawl (Killen, Nati, & Suski, 2015). These last studies were carried out in small experimental settings; thus, their applicability to more natural situations is unclear.

Exceptions that did look at differences in behaviour between captured fish and escapees in natural settings are Underwood, Winger, Fernö, and Engås's (2015) study on yellowtail flounder, *Limanda ferruginea*, in Newfoundland and Kim and Wardle's (2003) study on haddock, saithe, mackerel, cod and flatfish. Yellowtail flounders exhibited three different behavioural responses to an approaching trawl resulting in different catchability. Individuals that swam along the bottom in front of the trawl or rose gradually from the bottom exhibited higher capture rates, while individuals swimming directly upwards were captured less. This behaviour selectivity probably explains why swimming upwards is the most common response in yellowtail flounders (Underwood et al., 2015). According to Kim and Wardle's (2003) study, some individuals exhibited low variation in swimming speed, while others were characterized by large variations in velocity, showing a more erratic response. Kim and Wardle's (2003) study suggests that an erratic response close to the gear would allow escaping the net when there are large mesh sizes. This agrees with Killen et al.'s (2015) results for minnows in laboratory conditions. Trawl vulnerability seems to be associated with activity and swimming performance close to the gear. Individuals more likely to escape are those that swim close to the bottom with erratic movements (Table 1, Figure 1b).

Trawling might also lead to changes in habitat preference. Habitat‐specific fishing for Icelandic cod also seems to lead to behavioural changes in the population (Árnason, Hernandez, & Kristinsson, 2009; Jakobsdóttir et al., 2011). Trawl fishing pressure in shallow waters led to an increased abundance of individuals adapted to deep‐waters, opposing natural selection that otherwise would balance shallow‐water and deep‐water specializations (Jakobsdóttir et al., 2011); such fishing selection selects against specific genotypes (Árnason et al., 2009). Along similar lines, Northeast Arctic cod has shown a long‐term change towards more northern spawning habitats due to fishing mortality (Opdal & Jørgensen, 2015). Alós, Palmer, Linde‐Medina, and Arlinghaus (2014) showed that trawled individuals from two coastal fishes (*Diplodus annularis* and *Serranus scriba*) were shorter, deeper‐bodied and had smaller mouths than random. Hence, long and streamlined individuals should become more abundant in populations exposed to trawling, probably resulting also in more individuals with active swimming, higher swimming speeds and longer foraging searches (Alós et al., 2014).

Seining was originally considered to capture behavioural types nonselectively, but wild bluegill sunfish individuals seined from a lake were bolder than individuals captured by angling (Wilson et al., 2011). In contrast, pumpkinseed sunfish seined from an experimental pond were shyer relative to passively trapped individuals (Wilson et al., 1993). Moav and Wohlfarth (1970) concluded that vulnerability to being seined in carp was related to behavioural differences, more active individuals or those that tend to use the bottom of the ponds were less vulnerable to seining, but this idea was never directly tested.

## 4 Fishing indirect selection on behaviour and complex effects on multiple traits

**Figure 2:**
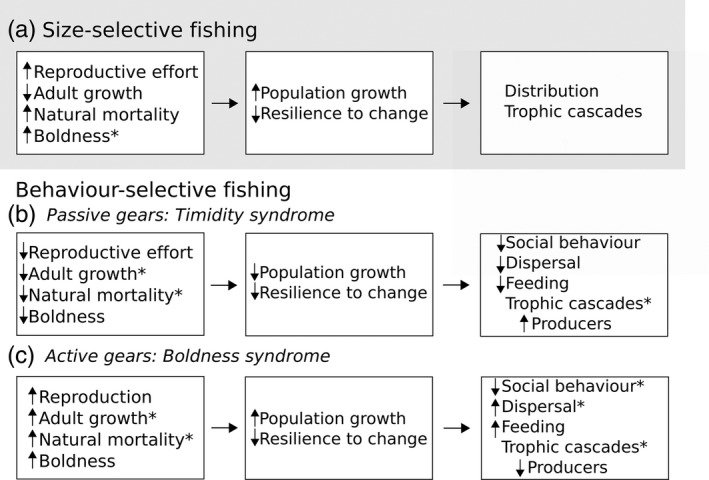
Summary of population and community level consequences of (a) size‐selective fishing, (b) passive and (c) active gear behaviour selection. Real world scenarios often involve multitrait responses that are context‐dependent that make it difficult to predict outcomes without considering the full suite of trait changes and specific contexts. * refers to cases where the general expectation is not met resulting in counterintuitive responses due to specific past evolutionary history and context dependency; see the text for examples.

Because behaviour is correlated with size and life‐history traits, laboratory experiments involving positive size‐selective fishing (e.g., selective removal of large individuals) induced widespread changes in behaviour: more social and timid guppies (Diaz Pauli et al., 2014), less exploratory individuals in zebra fish (*Danio rerio*; Uusi‐Heikkilä et al., 2015) and lower consumption rates and reduced willingness to forage (*Menidia menidia;* Walsh, Munch, Chiba, & Conover, 2006), compared to populations where small individuals were fished out (Figure 1d). These behavioural traits are associated with a slow pace of life (Réale et al., 2010), which conflicts with the usual notion of a short lifespan being associated with a fast pace of life and the expectation that fishing leads to boldness by devaluing the future (Figure 2a; Heino et al., 2013). Conflicting selection pressures and trait correlations that contradict the simple, pace of life view have also been observed in nonfishing contexts (Réale et al., 2010). Villegas‐Ríos et al. (2014) studied links between physiological state (reproductive vs. feeding), activity and catchability in natural conditions for the Ballan wrasse (*Labrus bergylta*). Intensely feeding and highly active individuals may be more likely to be captured by gill nets, while reproductive individuals tend to move less and thus have lower catchability (Villegas‐Ríos et al., 2014). Although this study correlated behavioural, physiological and catchability from different data sets, it may help to discern the vulnerability of individuals with different levels of activity, parental care and feeding within a population.

Several studies have quantified multitrait responses to fishing. Cooke, Suski, Ostrand, Wahl, and Philipp (2007) contrasted lines of largemouth bass selected for high versus low vulnerability to angling and found that males from the high vulnerability line (more aggressive and presumably bolder) were better at parental care relative to males from the low vulnerability line. Similar results were found for bluegill; lakes with high fishing pressure exhibited higher numbers of nonparental males compared to lakes with low fishing pressure (Drake, Claussen, Philipp, & Pereira, 1997). Largemouth bass from high angling vulnerability lines exhibited higher heart rate (Philipp et al., 2009), metabolic scope, more frequent startle responses with burst swimming rather than steady swimming (Redpath et al., 2010) and higher reproductive fitness (Sutter et al., 2012). Populations of largemouth bass subjected to angling are expected to respond to fishing by acquiring physiological and behavioural traits similar to those of the low vulnerability line, which depending on the context may result in populations with lower fitness and catchability, and diminishing population viability and quality of the recreational fishery (Sutter et al., 2012).

A similar complex trait response was found for vulnerability to seining by comparing two populations of carp. The populations experienced different selection pressures due to differences in their culture conditions. In China, individuals experienced high density and seine fishing, while individuals experienced no fishing pressure in Europe. Chinese individuals developed the ability to escape the seine (probably linked to higher activity; Moav & Wohlfarth, 1970), earlier maturity and slower adult growth, resulting in lower efficiency of the Chinese culture (Wohlfarth, Moav, & Hulata, 1975).

Thus, the simple expectation that direct selection from fishing should result in increased adaptation, and thus, higher fitness and viability might be violated when correlated characters exhibit maladaptive changes (e.g., reduced parental care, willingness to forage, larval viability and reproduction; Walsh et al., 2006; Sutter et al., 2012; Uusi‐Heikkilä et al., 2015). Understanding the multitrait response to fishing (of which behaviour is likely a key component) is thus of paramount importance for projecting impacts on fisheries success and on overall ecological dynamics.

## 5 Population, community, ecosystem and fisheries‐level consequences

In recent years, it has been acknowledged that rapid change in ecologically important traits can have major impacts on ecological dynamics (Schoener, 2011). The effect of size‐selective fishing on life‐history traits and its population level consequences are clear (Heino et al., 2013). But beyond the direct impacts on mortality rates and thus population, community and ecosystem dynamics, predator effects on prey traits (behaviour, physiology, morphology and life histories) also affect prey populations, species interactions and hence communities (Lima, 1998; Madin, Dill, Ridlon, Heithaus, & Warner, 2016). These phenotypic changes (plastic or evolutionary) likely have ecological consequences for the ecosystem and the fishery (Arlinghaus et al., 2016; Ward et al., 2016). However, both the behavioural change and the ecological consequences can be context‐specific (Palkovacs et al., 2012) and depend on past evolutionary history (Sih et al. 2011). In particular, fishing might result in counterintuitive responses (Pine, Martell, Walters, & Kitchell, 2009) if the multitrait response and the context dependency are not considered.

On an ontogenetic scale, exposure to predation risk, particularly early in life, can induce prey behavioural changes that persist over a lifetime (Lima, 1998). Exposure to predation risk in one generation can carry over to produce adaptive epigenetic effects on offspring personality (Stein & Bell, 2014). Thus, exposure to fishing gear might result in persistent, essentially fixed antipredator/antigear behaviours (e.g., high vigilance, low activity, staying near a refuge, living in schools) that can even carry over into future generations.

On an evolutionary timescale, given that personalities (i.e., consistent behaviour) are almost always heritable (Dochtermann, Schwab, & Sih, 2015), heavy personality‐dependent fishing pressure can drive the evolution of behaviour (e.g., Jakobsdóttir et al., 2011; Philipp et al., 2009). If, for example, passive gears tend to kill bold fish, this could drive the evolution of lower average boldness as seen for predator effects on prey in wild populations (Dingemanse et al., 2009) and perhaps lower variance in behavioural types (i.e., populations dominated by the less vulnerable behavioural type; Figure 1). Direct estimates of heritabilities for traits affected by fishing in the wild are still limited and can be affected by fishing selection (Killen, Adriaenssens, Marras, Claireaux, & Cooke, 2016). Predation can affect the variance components (additive genetic or residual) and heritability values of personality traits both at ontogenetic and evolutionary levels; for instance, it can lead to higher heritability values if it represents fluctuating selection leading to increased variance in the additive genetic component, or lower values if the selection is directional (Dingemanse et al., 2009)**.**

## 6 Population consequences

Fishing‐induced changes on behaviour likely affect numerous aspects of within‐species social and population dynamics. Several studies indeed show that fishing selection affects the fishes' social interactions, feeding rates, diets, intraspecific exploitative and interference competition, size‐dependent cannibalism rates, mating dynamics and parental care (Nannini, Wahl, Philipp, & Cooke, 2011; Sutter et al., 2012; Walsh et al., 2006), which in turn affects growth and recruitment. “Fast” personalities (bold, aggressive, exploratory, active) are typically associated with high metabolic rates and growth (Biro & Post, 2008) and fast life histories with early reproduction (Réale et al., 2010). Therefore, passive gear's selectivity would result in slow personalities and slow life history (Arlinghaus et al. 2016; Figure 2b), while active gears would result in a fast pace of life, similar to what is expected when only size selection is considered (Heino et al., 2013; Figure 2a,c). However, these relationships are context‐specific and such intuitive conclusions are not always met. For instance, with low food availability, largemouth bass with a “slow personality” and low metabolic rate grew faster than those of high metabolic rate and aggression (Sutter et al., 2012). In laboratory settings, populations that were positive size‐selected and hence with faster life histories exhibited lower reproductive output and timid behaviours (Diaz Pauli et al., 2014; Uusi‐Heikkilä et al., 2015; Walsh et al., 2006) contrary to expectations (Heino et al., 2013).

Fishing‐induced changes in the average (or variation) of a population's boldness could likely also affect the fishes' dispersal and range expansion (Harrison et al., 2015). Both passive and active gears seem to reduce dispersal and habitat range in cod and sockeye salmon (Jakobsdóttir et al., 2011; Olsen et al., 2012; Opdal & Jørgensen, 2015; Quinn et al., 2007), although trawling could also lead to larger habitat range (Alós et al., 2014). Personality‐dependent dispersal behaviour can, in principle, affect a broad range of aspects of spatial ecology including metapopulation/metacommunity dynamics, migratory success, disease spread and movement in and out of marine protected areas (Nilsson, Bronmark, Hansson, & Chapman, 2014).

Alterations in the composition of personality types in a population can also affect social interactions. Individual differences in behaviour and physiology determine the position of individuals within a group and its stability (Marras et al., 2014; Taborsky & Oliveira, 2012). Shy individuals are often more social compared to bold ones (Réale et al., 2010); hence, passive gear's selection can lead to more tight social groups relative to active gears. But if key individuals (i.e., movement leaders or knowledgeable demonstrators; Modlmeier, Keiser, Watters, Sih, & Pruitt, 2014) were generally bold ones, the opposite would occur (Arlinghaus et al., 2016). Diverse groups relative to homogenous ones seem to perform better at different collective behaviours (Dyer, Croft, Morrell, & Krause, 2009; Fischer, Bessert‐Nettelbeck, Kotrschal, & Taborsky, 2015). Thus, the selective removal of any type of individuals can disturb group stability and collective behaviour (Figure 2b,c). Because collective behaviour often underlies the dynamics of competition, mating, migration and social foraging, it can have major impacts on individual fitness and consequences at the population level (Taborsky & Oliveira, 2012).

## 7 Community, ecosystem and fisheries consequences

Fishing‐induced evolution of behaviour would also likely have important impacts on multiple species interactions, both at higher and lower trophic levels. In a simple three trophic level community (predator–consumer–producer) when fishing targets the middle species, it may affect traits that affect encounter rates with predators. Fast life histories can result in higher natural mortality when fishing is size‐selective (Jørgensen & Holt, 2013; Figure 2a), but it is not clear whether this holds when fishing selects directly on behaviour (Arlinghaus et al., 2016). If fishing causes fish to be less bold and aggressive, this would likely reduce their likelihood of being killed by natural predators (Sih, Cote, Evans, Fogarty, & Pruitt, 2012; Arlinghaus et al., 2016; Figure 2b), but the opposite would be true when fishing selection is associated with active gears and boldness is favoured (Figure 2c). The effects of fishing on risk from natural predators might depend on the match/mismatch between avoidance behaviour triggered by novel fishing gear as opposed to the natural predators' hunting mode (Sih et al., 2010). For example, passive gear that favours the survival of more timid, inactive fish might, as a by‐product, decreases predation by ambush predators that rarely encounter inactive prey (Arlinghaus et al., 2016), but might also reduce escape success from active, coursing predators, increasing predation.

Fishing can also affect the lowest trophic level (producers) by reducing the numbers of the target fish (consumers), thus reducing pressure on their food source (producers) and allowing the producers to become more abundant. But alterations in fish personality by active and passive gears can drive higher or lower feeding rates, respectively (Preisser, Bolnick, & Benard, 2005; Figure 2b,c), resulting in lower or higher abundances of producers. Moreover, fishing selection could also lead to changes in dietary preferences complicating this picture further. For instance, omnivorous guppies from high predation (HP) sites evolved not just faster life histories, but a tendency to consume more invertebrates and fewer algae than those that evolved under low predation (LP) pressure. Accordingly, mesocosms stocked with HP guppies had fewer invertebrates and higher algal standing stocks than those with LP guppies (Bassar et al., 2010).

Fishing targeting fish on a intermediate trophic level could potentially induce similar behavioural cascades, if fishing gears favour bold individuals (Diaz Pauli et al., 2015) or individuals with different feeding rates (Nannini et al., 2011; Walsh et al., 2006). However, if fishing targets predator (i.e., the highest trophic level rather than the middle), the consequences for the consumer and producer would be the opposite: increased abundance of consumers and decreased of producers, as described in Arlinghaus et al., 2016. Again, the magnitude of the cascading impacts on the overall food web could depend on the behavioural type, feeding rate and diet preferences of the target fish and on the complexity of the food web.

Finally, note that for some issues, in particular, ecological resilience, the effect of fishing on the maintenance of variation in personality might be more important than average personality per se. Reduced diversity can be associated with lower competitive ability and narrower resource utilization (Budaev & Brown, 2011), reduced population stability and viability and a decrease in the population's potential to adapt to changing environments to avoid extinction (Sih et al., 2012; Smith & Blumstein, 2013). In the case of exploited populations, these changes can reduce the potential for recovery. Selective fishing would then contribute to slow recovery of overexploited populations after fishing halts and the environment returns to natural conditions (Smith & Blumstein, 2013). This is expected for both size‐ and behaviour‐selective fishing (Figure 2; Heino et al., 2013).

Overall, this section shows the relevance of behavioural responses when evaluating the ecological impact of fishing gears expanding on Pine et al. (2009). For example, gill net fishing or introducing mixed minimum and maximum size limits are suggested to slow down fisheries‐induced evolution of maturation and boost yield (Matsumura, Arlinghaus, & Dieckmann, 2011; Zimmermann & Jørgensen, 2014), while pot fishery is considered to be relatively benign because of its low by‐catch and low impact on the ecosystem (Blyth, Kaiser, Edwards‐Jones, & Hart, 2004; FAO 2003). Although we do not disagree on the benefits of using pots and gill net instead of trawling, their behaviour and size selectivity and their consequences for diversity and viability should be considered to make more informed recommendations that take into account long‐term impacts of fishing on the ecosystem. Comparable reductions in fisheries‐induced evolution on maturation obtained with gill net dome‐shaped selectivity on size could be obtained using mixed‐gear fisheries that results in lower selectivity towards any particular behavioural type. Similar considerations should be taken into account while evaluating the benefit of no‐take protected areas. These may mitigate the effect of selective fishing on behaviour (Twardek et al. in press) or may only favour shy individuals or individuals with small home ranges, while bold active ones are fished when they disperse outside the reserve, which could ultimately bias stock assessments (Alós, Puiggrós et al., 2015; Villegas‐Ríos, Moland, & Olsen, 2016). Whether this is the ideal way of maintaining behavioural diversity or of producing two different populations with low genetic diversity remains to be tested.

## 8 Future experiments

At this stage, much of what we know about the selective effect of fishing on behaviour and its consequences comes from few studies whose results are highly context‐dependent. Thus, drawing general conclusions are speculative at this time and more research is needed. We suggest that three different fronts should be considered:

1. Assessing the differences in behaviour between captured individuals and escapees in natural conditions would improve our understanding on the behavioural selectivity of fishing. Particularly interesting would be to apply experimental designs such as those of Huse and Vold (2010), Marçalo et al. (2013) and Ingólfsson and Jørgensen (2006) that can retain both captured individuals and escapees from active gears, where data are most limited. Then, the combined use of laboratory experiments and telemetry in natural conditions would allow assessing behavioural, physiological and life‐history differences between groups and the relative importance of different traits in gear selection in the wild. This could be complemented with observations via underwater cameras or sonar during the capture process similar to those of Rosen et al. (2012) and Underwood et al. (2015). These set‐ups should allow us to assess the consequences of specific and natural drivers of change in a controlled way (as shown for passive gears for instance in Olsen et al., 2012; Alós, Palmer, et al., 2015) and move us away from relying on laboratory set‐ups where the selective pressures may be altered or simplified in unnatural conditions.
2. Estimates of heritability of behaviour are necessary to establish whether fishing‐induced evolution of behaviour is taking place. These ideally should be obtained from natural conditions where the selection takes place using pedigree data or through laboratory‐reared second‐generation offspring to wild‐caught parent regression, although these estimates remain challenging. Estimates of behavioural repeatability (which may set the upper level for heritability; Dochtermann et al., 2015; Killen et al., 2016) in the wild as in Olsen et al. (2012) or estimates of heritability of behaviour linked to fishing selection in laboratory conditions (Philipp et al., 2009) could be a good start.
3. Studies looking at whether escapee‐only populations lead to different cascading effects in the ecosystem relative to captured‐only populations or populations with mixed natural distribution of traits would greatly improve our knowledge on consequences of selective fishing. Here, the contrast of captured and escaped fish should not be limited to behaviour‐selected traits, but any type of selection subjected by fishing. This type of study could involve laboratory selection experiments that create different lines representing escapees, captured and mixed phenotypes. The different lines would be then introduced to mesocosms with simplified but diverse communities to contrast their effects on overall community dynamics, including multispecies effects such as trophic cascades.

## References

[eva12456-bib-0001] Alós, J. , Palmer, M. , Linde‐Medina, M. , & Arlinghaus, R. (2014). Consistent size‐independent harvest selection on fish body shape in two recreationally exploited marine species. Ecology and Evolution, 4, 2154–2164. 25360257 10.1002/ece3.1075 PMC4201430

[eva12456-bib-0002] Alós, J. , Palmer, M. , Trías, P. , Díaz‐Gil, C. , & Arlinghaus, R. (2015). Recreational angling intensity correlates with alteration of vulnerability to fishing in a carnivorous coastal fish species. Canadian Journal of Fisheries and Aquatic Sciences, 72, 217–225.

[eva12456-bib-0003] Alós, J. , Puiggrós, A. , Díaz‐Gil, C. , Palmer, M. , Rosselló, R. , & Arlinghaus, R. (2015). Empirical evidence for species‐specific export of fish naïveté from a no‐take marine protected area in a coastal recreational hook and line fishery. PLoS One, 10, e0135348. 26275290 10.1371/journal.pone.0135348 PMC4537300

[eva12456-bib-0004] Arlinghaus, R. , Laskowski, K. L. , Alós, J. , Klefoth, T. , Monk, C. T. , Nakayama, S. , & Schröder, A. (2016). Passive gear‐induced timidity syndrome in wild fish populations and its potential ecological and managerial implications. Fish and Fisheries. doi:10.1111/faf.12176.

[eva12456-bib-0005] Árnason, E. , Hernandez, U. B. , & Kristinsson, K. (2009). Intense habitat‐specific fisheries‐induced selection at the molecular *Pan* I locus predicts imminent collapse of a major cod fishery. PLoS One, 4, e5529. 19479037 10.1371/journal.pone.0005529 PMC2682699

[eva12456-bib-0006] Bassar, R. D. , Marshall, M. C. , López‐Sepulcre, A. , Zandonà, E. , Auer, S. K. , Travis, J. , … Post, E. (2010). Local adaptation in Trinidadian guppies alters ecosystem processes. Proceedings of the National Academy of Sciences of the United States of America, 107, 3616–3621. 20133670 10.1073/pnas.0908023107 PMC2840427

[eva12456-bib-0007] Bergseth, B. J. , Williamson, D. H. , Frisch, A. J. , & Russ, G. R. (2016). Protected areas preserve natural behaviour of a targeted fish species on coral reefs. Biological Conservation, 198, 202–209.

[eva12456-bib-0008] Biro, P. A. , & Post, J. R. (2008). Rapid depletion of genotypes with fast growth and bold personality traits from harvested fish populations. Proceedings of the National Academy of Sciences of the United States of America, 105, 2919–2922. 18299567 10.1073/pnas.0708159105 PMC2268560

[eva12456-bib-0009] Blyth, R. E. , Kaiser, M. J. , Edwards‐Jones, G. , & Hart, P. J. B. (2004). Implications of a zoned fishery management system for marine benthic communities. Journal of Applied Ecology, 41, 951–961.

[eva12456-bib-0010] Brown, C. , & Warburton, K. (1999a). Differences in timidity and escape responses between predator‐naive and predator‐sympatric rainbowfish populations. Ethology, 105, 491–502.

[eva12456-bib-0011] Brown, C. , & Warburton, K. (1999b). Social mechanisms enhance escape responses in shoals of rainbowfish, *Melanotaenia duboulayi* . Environmental Biology of Fishes, 56, 455–459.

[eva12456-bib-0012] Budaev, S. , & Brown, C. (2011). Personality traits and behaviour In Brown C., Laland K., & Krause J. (Eds.), Fish cognition and behavior, 2nd ed. (pp. 135–165). Oxford: Blackwell Publishing Ltd..

[eva12456-bib-0013] Candolin, U. , & Wong, B. B. M. (2012). Behavioural responses to a changing world. Mechanisms and consequences (Candolin U., & Wong B. B. M.) (pp. 1–270). Oxford: Oxford University Press.

[eva12456-bib-0014] Chapman, B. B. , Hulthén, K. , Blomqvist, D. R. , Hansson, L.‐A. , Nilsson, J.‐Å. , Brodersen, J. , … Brönmark, C. (2011). To boldly go: Individual differences in boldness influence migratory tendency. Ecology Letters, 14, 871–876. 21718420 10.1111/j.1461-0248.2011.01648.x

[eva12456-bib-0015] Conover, D. O. , & Munch, S. B. (2002). Sustaining fisheries yields over evolutionary time scales. Science, 297, 94–96. 12098697 10.1126/science.1074085

[eva12456-bib-0016] Cooke, S. J. , Suski, C. D. , Ostrand, K. G. , Wahl, D. H. , & Philipp, D. P. (2007). Physiological and behavioral consequences of long‐term artificial selection for vulnerability to recreational angling in a teleost fish. Physiological and Biochemical Zoology, 80, 480–490. 17717811 10.1086/520618

[eva12456-bib-0017] Diaz Pauli, B. , Utne‐Palm, A. C. , Wiech, M. , Ehlman, S. , Sih, A. , & Heino, M. (2014). Does fishing affect behaviour? An experimental test with guppy (Poecilia reticulata) populations. ICES CM 2014/E18.

[eva12456-bib-0018] Diaz Pauli, B. , Wiech, M. , Heino, M. , & Utne‐Palm, A. C. (2015). Opposite selection on behavioural types by active and passive fishing gears in a simulated guppy *Poecilia reticulata* fishery. Journal of Fish Biology, 86, 1030–1045. 25619538 10.1111/jfb.12620

[eva12456-bib-0019] Dingemanse, N. J. , Van der Plas, F. , Wright, J. , Réale, D. , Schrama, M. , Roff, D. A. , … Barber, I. (2009). Individual experience and evolutionary history of predation affect expression of heritable variation in fish personality and morphology. Proceedings of the Royal Society B: Biological Sciences, 276, 1285–1293. 19129142 10.1098/rspb.2008.1555 PMC2660958

[eva12456-bib-0020] Dochtermann, N. A. , Schwab, T. , & Sih, A. (2015). The contribution of additive genetic variation to personality variation: Heritability of personality. Proceedings of the Royal Society B: Biological Sciences, 282, 20142201. 25392476 10.1098/rspb.2014.2201 PMC4262176

[eva12456-bib-0021] Drake, M. T. , Claussen, J. E. , Philipp, D. P. , & Pereira, D. L. (1997). A comparison of bluegill reproductive strategies and growth among lakes with different fishing intensities. North American Journal of Fisheries Management, 17, 496–507.

[eva12456-bib-0022] Dyer, J. R. G. , Croft, D. P. , Morrell, L. J. , & Krause, J. (2009). Shoal composition determines foraging success in the guppy. Behavioural Ecology, 20, 165–171.

[eva12456-bib-0023] Edeline, E. , Carlson, S. M. , Stige, L. C. , Winfield, I. J. , Fletcher, J. M. , James, J. B. , … Stenseth, N. C. (2007). Trait changes in a harvested population are driven by a dynamic tug‐of‐war between natural and harvest selection. Proceedings of the National Academy of Sciences of the United States of America, 104, 15799–15804. 17898170 10.1073/pnas.0705908104 PMC2000386

[eva12456-bib-0024] Engås, A. , & Godø, O. R. (1989). Escape of fish under the fishing line of a Norwegian sampling trawl and its influence on survey results. ICES Journal of Marine Science, 45, 269–276.

[eva12456-bib-0025] FAO , Food and Agriculture Organisation of the United Nations. 2003 The ecosystem approach to fisheries. In FAO Technical Guidelines for Responsible Fisheries. Suppl. 2. FAO, Rome.

[eva12456-bib-0026] Fischer, S. , Bessert‐Nettelbeck, M. , Kotrschal, A. , & Taborsky, B. (2015). Rearing‐group size determines social competence and brain structure in a cooperatively breeding cichlid. The American Naturalist, 186, 123–140. 10.1086/681636 26098344

[eva12456-bib-0027] Fraser, D. F. , Gilliam, J. F. , Daley, M. J. , Le, A. N. , & Skalski, G. T. (2001). Explaining leptokurtic movement distributions: Intrapopulation variation in boldness and exploration. The American Naturalist, 158, 124–135. 10.1086/321307 18707341

[eva12456-bib-0028] Gabriel O., Lange K., Dahm E., & Wendt T. (Eds.) (2005). Fish catching methods of the world. Oxford: John Wiley & Sons.

[eva12456-bib-0029] Halpern, B. S. , Walbridge, S. , Selkoe, K. A. , Kappel, C. V. , Micheli, F. , D'Agrosa, C. , … Watson, R. (2008). A global map of human impact on marine ecosystems. Science, 319, 948–952. 18276889 10.1126/science.1149345

[eva12456-bib-0030] Handegard, N. O. , & Tjøstheim, D. (2005). When fish meet a trawling vessel: Examining the behaviour of gadoids using a free‐floating buoy and acoustic split‐beam tracking. Canadian Journal of Fisheries and Aquatic Sciences, 62, 2409–2422.

[eva12456-bib-0031] Härkönen, L. , Hyvärinen, P. , Niemelä, P. T. , & Vainikka, A. (2015). Behavioural variation in Eurasian perch populations with respect to relative catchability. Acta Ethologica, 19, 21–31.

[eva12456-bib-0032] Härkönen, L. , Hyvärinen, P. , Paappanen, J. , Vainikka, A. , & Tierney, K. (2014). Explorative behavior increases vulnerability to angling in hatchery‐reared brown trout (*Salmo trutta*). Canadian Journal of Fisheries and Aquatic Sciences, 71, 1900–1909.

[eva12456-bib-0033] Harrison, P. M. , Gutowsky, L. F. G. , Martins, E. G. , Patterson, D. A. , Cooke, S. J. , & Power, M. (2015). Personality‐dependent spatial ecology occurs independently from dispersal in wild burbot (*Lota lota*). Behavioural Ecology, 26, 483–492.

[eva12456-bib-0034] Heino, M. , Baulier, L. , Boukal, D. S. , Ernande, B. , Johnston, F. D. , Mollet, F. M. , … Dieckmann, U. (2013). Can fisheries‐induced evolution shift reference points for fishery management? ICES Journal of Marine Science, 70, 707–721.

[eva12456-bib-0035] Heino, M. , Diaz Pauli, B. , & Dieckmann, U. (2015). Fisheries‐induced evolution. Annual Review of Ecology, Evolution, and Systematics, 46, 461–480.

[eva12456-bib-0036] Heino, M. , & Godø, O. R. (2002). Fisheries‐induced selection pressures in the context of sustainable fisheries. Bulletin of Marine Science, 70, 639–656.

[eva12456-bib-0037] Heino, M. , Porteiro, F. M. , Sutton, T. T. , Falkenhaug, T. , Godø, O. R. , & Piatkowski, U. (2011). Catchability of pelagic trawls for sampling deep‐living nekton in the mid‐North Atlantic. ICES Journal of Marine Science, 68, 377–389.

[eva12456-bib-0038] Hendry, A. P. , Farrugia, T. J. , & Kinnison, M. T. (2008). Human influences on rates of phenotypic change in wild animal populations. Molecular Ecology, 17, 20–29. 18173498 10.1111/j.1365-294X.2007.03428.x

[eva12456-bib-0039] Hunter, J. R. , & Wisby, W. J. (1964). Net avoidance behavior of carp and other species of fish. Journal of the Fisheries Board of Canada, 21, 613–633.

[eva12456-bib-0040] Huse, I. , & Vold, A. (2010). Mortality of mackerel (*Scomber scombrus* L.) after pursing and slipping from a purse seine. Fisheries Research, 106, 54–59.

[eva12456-bib-0041] Ingólfsson, Ó. A. , & Jørgensen, T. (2006). Escapement of gadoid fish beneath a commercial bottom trawl: Relevance to the overall trawl selectivity. Fisheries Research, 79, 303–312.

[eva12456-bib-0042] Jakobsdóttir, K. B. , Pardoe, H. , Magnússon, Á. , Björnsson, H. , Pampoulie, C. , Ruzzante, D. E. , & Marteinsdóttir, G. (2011). Historical changes in genotypic frequencies at the Pantophysin locus in Atlantic cod (*Gadus morhua*) in Icelandic waters: Evidence of fisheries‐induced selection? Evolutionary Applications, 4, 562–573. 25568005 10.1111/j.1752-4571.2010.00176.x PMC3352422

[eva12456-bib-0043] Januchowski‐Hartley, F. A. , Graham, N. A. J. , Cinner, J. E. , & Russ, G. R. (2015). Local fishing influences coral reef fish behavior inside protected areas of the Indo‐Pacific. Biological Conservation, 182, 8–12.

[eva12456-bib-0044] Jørgensen, C. , & Holt, R. E. (2013). Natural mortality: Its ecology, how it shapes fish life histories, and why it may be increased by fishing. Journal of Sea Research, 75, 8–18.

[eva12456-bib-0045] Kekäläinen, J. , Podgorniak, T. , Puolakka, T. , Hyvärinen, P. , & Vainikka, A. (2014). Individually assessed boldness predicts *Perca fluviatilis* behaviour in shoals, but is not associated with the capture order or angling method. Journal of Fish Biology, 85, 1603–1616. 25270290 10.1111/jfb.12516

[eva12456-bib-0046] Kelleher, K. (2005). Discards in the world's marine fisheries. An update. FAO Fisheries Technical Paper. No. 470. Rome, FAO.

[eva12456-bib-0047] Killen, S. S. , Adriaenssens, B. , Marras, S. , Claireaux, G. , & Cooke, S. J. (2016). Context dependency of trait repeatability and its relevance for management and conservation of fish populations. Conservation Physiology, 4, cow007. 10.1093/conphys/cow007 PMC4922260 27382470

[eva12456-bib-0048] Killen, S. S. , Nati, J. J. H. , & Suski, C. D. (2015). Vulnerability of individual fish to capture by trawling is influenced by capacity for anaerobic metabolism. Proceedings of the Royal Society B: Biological Sciences, 282, 1–25. 10.1098/rspb.2015.0603 PMC4632608 26246542

[eva12456-bib-0049] Kim, Y.‐H. , & Wardle, C. S. (2003). Optomotor response and erratic response: Quantitative analysis of fish reaction to towed fishing gears. Fisheries Research, 60, 455–470.

[eva12456-bib-0050] Klefoth, T. , Skov, C. , Krause, J. , & Arlinghaus, R. (2012). The role of ecological context and predation risk‐stimuli in revealing the true picture about the genetic basis of boldness evolution in fish. Behavioural Ecology and Sociobiology, 66, 547–559.

[eva12456-bib-0051] Lima, S. L. (1998). Stress and decision‐making under the risk of predation: Recent developments from behavioral, reproductive, and ecological perspectives. Advances in the Study of Behaviour, 27, 215–290.

[eva12456-bib-0052] Madin, E. M. P. , Dill, L. M. , Ridlon, A. D. , Heithaus, M. R. , & Warner, R. R. (2016). Human activities change marine ecosystems by altering predation risk. Global Change Biology, 22, 44–60. 26448058 10.1111/gcb.13083

[eva12456-bib-0053] Marçalo, A. , Araújo, J. , Pousão‐Ferreira, P. , Pierce, G. J. , Stratoudakis, Y. , & Erzini, K. (2013). Behavioural responses of sardines *Sardina pilchardusto* simulated purse‐seine capture and slipping. Journal of Fish Biology, 83, 480–500. 23991869 10.1111/jfb.12184

[eva12456-bib-0054] Marras, S. , Killen, S. S. , Lindström, J. , McKenzie, D. J. , Steffensen, J. F. , & Domenici, P. (2014). Fish swimming in schools save energy regardless of their spatial position. Behavioural Ecology and Sociobiology, 69, 219–226. 10.1007/s00265-014-1834-4 PMC4293471 25620833

[eva12456-bib-0055] Matsumura, S. , Arlinghaus, R. , & Dieckmann, U. (2011). Assessing evolutionary consequences of size‐selective recreational fishing on multiple life‐history traits, with an application to northern pike (*Esox lucius*). Evolutionary Ecology, 25, 711–735.

[eva12456-bib-0056] Miller, R. B. (1957). Have the genetic patterns of fishes been altered by introductions or by selective fishing? Journal of the Fisheries Research Board of Canada, 14, 797–806.

[eva12456-bib-0057] Misund, O. A. (1990). Sonar observations of schooling herring: School dimensions, swimming behaviour, and avoidance of vessel and purse seine. Rapports et Procès‐Verbaux des Réunions du Conseil Permanent International pour l'Exploration de la Mer, 189, 135–146.

[eva12456-bib-0058] Moav, R. , & Wohlfarth, G. W. (1970). Genetic correlation between seine escapability and growth capacity in carp. Journal of Heredity, 61, 153–157. 5480949 10.1093/oxfordjournals.jhered.a108067

[eva12456-bib-0059] Modlmeier, A. P. , Keiser, C. N. , Watters, J. V. , Sih, A. , & Pruitt, J. N. (2014). The keystone individual concept: An ecological and evolutionary overview. Animal Behaviour, 89, 53–62.

[eva12456-bib-0060] Nannini, M. A. , Wahl, D. H. , Philipp, D. P. , & Cooke, S. J. (2011). The influence of selection for vulnerability to angling on foraging ecology in largemouth bass *Micropterus salmoides* . Journal of Fish Biology, 79, 1017–1028. 21967587 10.1111/j.1095-8649.2011.03079.x

[eva12456-bib-0061] Nilsson, J.‐Å. , Bronmark, C. , Hansson, L. A. , & Chapman, B. B. (2014). Individuality in movement: The role of animal personality In Hansson L. A., & Akesson S. (Eds.), Animal movement across scales (pp. 90–109). Oxford, UK: Oxford University Press.

[eva12456-bib-0062] Olsen, E. M. , Heupel, M. R. , Simpfendorfer, C. A. , & Moland, E. (2012). Harvest selection on Atlantic cod behavioral traits: Implications for spatial management. Ecology and Evolution, 2, 1549–1562. 22957161 10.1002/ece3.244 PMC3434912

[eva12456-bib-0063] Opdal, A. F. , & Jørgensen, C. (2015). Long‐term change in a behavioural trait: Truncated spawning distribution and demography in Northeast Arctic cod. Global Change Biology, 21, 1521–1530. 25336028 10.1111/gcb.12773 PMC4404994

[eva12456-bib-0064] Ovegård, M. , Berndt, K. , & Lunneryd, S. G. (2012). Condition indices of Atlantic cod (*Gadus morhua*) biased by capturing method. ICES Journal of Marine Science, 69, 1781–1788.

[eva12456-bib-0065] Özbilgin, H. , & Glass, C. (2004). Role of learning in mesh penetration behaviour of haddock (*Melanogrammus aeglefinus*). ICES Journal of Marine Science, 61, 1190–1194.

[eva12456-bib-0066] Palkovacs, E. P. , Kinnison, M. T. , Correa, C. , Dalton, C. M. , & Hendry, A. P. (2012). Fates beyond traits: Ecological consequences of human‐induced trait change. Evolutionary Applications, 5, 183–191. 25568040 10.1111/j.1752-4571.2011.00212.x PMC3353338

[eva12456-bib-0067] Philipp, D. P. , Cooke, S. J. , Claussen, J. E. , Koppelman, J. , Suski, C. D. , & Burkett, D. (2009). Selection for vulnerability to angling in largemouth bass. Transactions of the American Fisheries Society, 138, 189–199.

[eva12456-bib-0068] Pine, W. E. III , Martell, S. , Walters, C. J. , & Kitchell, J. F. (2009). Counterintuitive responses of fish populations to management actions: Some common causes and implications for predictions based on ecosystem modeling. Fisheries, 34, 165–180.

[eva12456-bib-0069] Preisser, E. L. , Bolnick, D. I. , & Benard, M. F. (2005). Scared to death? The effects of intimidation and consumption in predator‐prey interactions. Ecology, 86, 501–509.

[eva12456-bib-0070] Pyanov, A. I. (1993). Fish learning in response to trawl fishing. ICES Marine Science Symposia, 196, 12–16.

[eva12456-bib-0071] Quinn, T. P. , Hodgson, S. , Flynn, L. , Hilborn, R. , & Rogers, D. E. (2007). Directional selection by fisheries and the timing of sockeye salmon (*Oncorhynchus nerka*) migrations. Ecological Applications, 17, 731–739. 17494392 10.1890/06-0771

[eva12456-bib-0072] Réale, D. , Garant, D. , Humphries, M. M. , Bergeron, P. , Careau, V. , & Montiglio, P. O. (2010). Personality and the emergence of the pace‐of‐life syndrome concept at the population level. Philosophical Transactions of the Royal Society B: Biological Sciences, 365, 4051–4063. 10.1098/rstb.2010.0208 PMC2992747 21078657

[eva12456-bib-0073] Redpath, T. D. , Cooke, S. J. , Suski, C. D. , Arlinghaus, R. , Couture, P. , Wahl, D. H. , & Philipp, D. P. (2010). The metabolic and biochemical basis of vulnerability to recreational angling after three generations of angling‐induced selection in a teleost fish. Canadian Journal of Fisheries and Aquatic Sciences, 67, 1983–1992.

[eva12456-bib-0074] Rosen, S. , Engås, A. , Fernö, A. , & Jørgensen, T. (2012). The reactions of shoaling adult cod to a pelagic trawl: Implications for commercial trawling. ICES Journal of Marine Science, 69, 303–312.

[eva12456-bib-0075] Schoener, T. W. (2011). The newest synthesis: Understanding the interplay of evolutionary and ecological dynamics. Science, 331, 426–429. 21273479 10.1126/science.1193954

[eva12456-bib-0076] Sih, A. , Bell, A. M. , & Johnson, J. C. (2004). Behavioral syndromes: An ecological and evolutionary overview. Trends in Ecology & Evolution, 19, 372–378. 16701288 10.1016/j.tree.2004.04.009

[eva12456-bib-0077] Sih, A. , Bolnick, D. I. , Luttbeg, B. , Orrock, J. L. , Peacor, S. D. , Pintor, L. M. , … Vonesh, J. R. (2010). Predator‐prey naïveté, antipredator behavior, and the ecology of predator invasions. Oikos, 119, 610–621.

[eva12456-bib-0078] Sih, A. , Cote, J. , Evans, M. , Fogarty, S. , & Pruitt, J. (2012). Ecological implications of behavioural syndromes. Ecology Letters, 15, 278–289. 22239107 10.1111/j.1461-0248.2011.01731.x

[eva12456-bib-0079] Sih, A. , Ferrari, M. C. O. , & Harris, D. J. (2011). Evolution and behavioural responses to human‐induced rapid environmental change. Evolutionary Applications, 4, 367–387. 25567979 10.1111/j.1752-4571.2010.00166.x PMC3352552

[eva12456-bib-0080] Smith, B. R. , & Blumstein, D. T. (2013). Animal personalities and conservation biology In Carere C., & Maestripieri D. (Eds.), Animal personalities: Behavior, physiology, and evolution (pp. 379–411). Chicago, IL: The University of Chicago Press.

[eva12456-bib-0081] Stein, L. R. , & Bell, A. M. (2014). Paternal programming in sticklebacks. Animal Behaviour, 95, 165–171. 27011391 10.1016/j.anbehav.2014.07.010 PMC4801484

[eva12456-bib-0082] Suski, C. D. , & Philipp, D. P. (2004). Factors affecting the vulnerability to angling of nesting male largemouth and smallmouth bass. Transactions of the American Fisheries Society, 133, 1100–1106.

[eva12456-bib-0083] Sutter, D. A. H. , Suski, C. D. , Philipp, D. P. , Klefoth, T. , Wahl, D. H. , Kersten, P. , … Arlinghaus, R. (2012). Recreational fishing selectively captures individuals with the highest fitness potential. Proceedings of the National Academy of Sciences of the United States of America, 109, 20960–20965. 23213220 10.1073/pnas.1212536109 PMC3529059

[eva12456-bib-0084] Taborsky, B. , & Oliveira, R. F. (2012). Social competence: An evolutionary approach. Trends in Ecology & Evolution, 27, 679–688. 23040461 10.1016/j.tree.2012.09.003

[eva12456-bib-0085] Tsuboi, J. , Morita, K. , Klefoth, T. , Endou, S. , & Arlinghaus, R. (2016). Behaviour‐mediated alteration of positively size‐dependent vulnerability to angling in response to historical fishing pressure in a freshwater salmonid. Canadian Journal of Fisheries and Aquatic Sciences, 73, 461–468.

[eva12456-bib-0086] Tuomainen, U. , & Candolin, U. (2011). Behavioural responses to human‐induced environmental change. Biological Reviews, 86, 640–657. 20977599 10.1111/j.1469-185X.2010.00164.x

[eva12456-bib-0087] Twardek, W. M. , Elvidge, C. K. , Wilson, A. D. M. , Algera, D. A. , Zolderdo, A. , Lougheed, S. C. , & Cooke, S. J. (2017). Do protected areas mitigate the effects of fisheries‐induced evolution on parental care behaviour of a teleost fish? Aquatic Conservation. doi: 10.1002/aqc.2718.

[eva12456-bib-0088] Underwood, M. , Winger, P. D. , Fernö, A. , & Engås, A. (2015). Behavior‐dependent selectivity of yellowtail flounder (*Limanda ferruginea*) in the mouth of a commercial bottom trawl. Fishery Bulletin, 113, 430–441.

[eva12456-bib-0089] Uusi‐Heikkilä, S. , Whiteley, A. R. , Kuparinen, A. , Matsumura, S. , Venturelli, P. A. , Wolter, C. , … Arlinghaus, R. (2015). The evolutionary legacy of size‐selective harvesting extends from genes to populations. Evolutionary Applications, 8, 597–620. 26136825 10.1111/eva.12268 PMC4479515

[eva12456-bib-0090] Uusi‐Heikkilä, S. , Wolter, C. , Klefoth, T. , & Arlinghaus, R. (2008). A behavioral perspective on fishing‐induced evolution. Trends in Ecology & Evolution, 23, 419–421. 18582988 10.1016/j.tree.2008.04.006

[eva12456-bib-0091] Vainikka, A. , Tammela, I. , & Hyvärinen, P. (2016). Does boldness explain vulnerability to angling in Eurasian perch *Perca fluviatilis*? Current Zoology, 62, 109–115. 10.1093/cz/zow003 PMC5804226 29491897

[eva12456-bib-0092] Villegas‐Ríos, D. , Alós, J. , Palmer, M. , Lowerre‐Barbieri, S. K. , Bañón, R. , Alonso‐Fernández, A. , & Saborido‐Rey, F. (2014). Life‐history and activity shape catchability in a sedentary fish. Marine Ecology Progress Series, 515, 239–250.

[eva12456-bib-0093] Villegas‐Ríos, D. , Moland, E. , & Olsen, E. M. (2016). Potential of contemporary evolution to erode fishery benefits from marine reserves. Fish and Fisheries. doi:10.1111/faf.12188

[eva12456-bib-0094] Walsh, S. J. (1992). Size‐dependent selection at the footgear of a groundfish survey trawl. North American Journal of Fisheries Management, 12, 625–633.

[eva12456-bib-0095] Walsh, M. R. , Munch, S. B. , Chiba, S. , & Conover, D. O. (2006). Maladaptive changes in multiple traits caused by fishing: Impediments to population recovery. Ecology Letters, 9, 142–148. 16958879 10.1111/j.1461-0248.2005.00858.x

[eva12456-bib-0096] Ward, T. D. , Algera, D. A. , Gallagher, A. J. , Hawkins, E. , Horodysky, A. , Jørgensen, C. , … Cooke, S. J. (2016). Understanding the individual to implement the ecosystem approach to fisheries management. Conservation Physiology, 4, cow005. 10.1093/conphys/cow005 PMC4825417 27293757

[eva12456-bib-0097] Wilson, A. D. M. , Binder, T. R. , McGrath, K. P. , Cooke, S. J. , Godin, J.‐G. J. , & Kraft, C. (2011). Capture technique and fish personality: Angling targets timid bluegill sunfish, *Lepomis macrochirus* . Canadian Journal of Fisheries and Aquatic Sciences, 68, 749–757.

[eva12456-bib-0098] Wilson, D. S. , Coleman, K. , Clark, A. B. , & Biederman, L. (1993). Shy‐bold continuum in pumpkinseed sunfish (*Lepomis gibbosus*): An ecological study of a psychological trait. Journal of Comparative Psychology, 107, 250–260.

[eva12456-bib-0099] Wohlfarth, G. , Moav, R. , & Hulata, G. (1975). Genetic differences between the Chinese and European races of the common carp. II. Multi‐character variation‐a response to the diverse methods of fish cultivation in Europe and China. Heredity, 34, 341–350. 1056321 10.1038/hdy.1975.43

[eva12456-bib-0100] Wong, B. B. M. , & Candolin, U. (2015). Behavioral responses to changing environments. Behavioural Ecology, 26, 665–667.

[eva12456-bib-0101] Zimmermann, F. , & Jørgensen, C. (2014). Influence of gear selectivity on FIE and yield. ICES CM 2014/E22.

